# Responding to structural inequities: Coping strategies among immigrant women during COVID-19

**DOI:** 10.1016/j.ssmmh.2023.100293

**Published:** 2023-12-24

**Authors:** Tara F. Abularrage, Heather M. Wurtz, Goleen Samari

**Affiliations:** a Heilbrunn Department of Population and Family Health, Mailman School of Public Health, Columbia University, New York, NY, USA; b Anthropology Department, University of Connecticut, Storrs, CT, USA; c Research Program on Global Health & Human Rights, Human Rights Institute, University of Connecticut, Storrs, CT, USA; d Population Studies and Training Center, Brown University, Providence, RI, USA; e Department of Population and Public Health Sciences, University of Southern California, Los Angeles, CA, USA

**Keywords:** Immigrants, Structural racism, Coping, Structural inequities, COVID-19, Gender

## Abstract

Examining coping strategies and resilience among immigrant communities reflects a commitment to working with immigrant communities to understand their needs while also identifying and building upon their strengths. In the United States, the physical, emotional, and economic impacts of the COVID-19 pandemic intersected with existing structural inequities to produce distinct challenges and stressors related to the pandemic, immigration, caregiving responsibilities, and structural xenophobia. Leveraging an understanding of the multilevel effects of stress, this qualitative study explores individual, interpersonal, and community-level coping strategies immigrant women used to respond to, alleviate, or reduce distress related to these compounding stressors. Using semi-structured in-depth interviews conducted in 2020 and 2021 with 44 first- and second-generation cisgender immigrant women from different national origins and 19 direct service providers serving immigrant communities in New York City, data were coded and analyzed using a constant comparative approach. Four central themes were identified: caregiving as a source of strength, leveraging resources, social connections, and community support. While women described a range of coping strategies they used to manage stressors and challenges, perspectives from direct service providers also connect these coping strategies to the harm-generating institutions, policies, and structures that produce and uphold structural oppression and inequities. Accounts from service providers point to the detrimental long-term effects of prolonged coping, underscoring a duality between resilience and vulnerability. Exploring the coping strategies cisgender immigrant women used to ease distress and promote resilience during a period of heightened structural vulnerability is critical to centering the experiences of immigrant women while simultaneously directing attention towards addressing the fundamental causes of cumulative disadvantage and the systems and structures through which it is transmitted.

## 1. Introduction

Immigrants constitute a significant portion of the United States (U.S.) population, accounting for approximately 14% (45.3 million people) (Esterline and Batalova, 2022). A growing body of scholarship has established the role of immigration as a social determinant of health, demonstrating how macro-level social factors that are produced and reproduced by social structures, policies, and institutions impact the health and well-being of immigrant communities (Castañeda et al., 2015). Moreover, immigrant populations often face “syndemic vulnerability,” wherein upstream social, economic, political, and structural determinants put them at risk of concurrent and deleteriously interacting forms of psychosocial stress and health adversity (McKnight-Eily, 2021; Sangaramoorthy and Carney, 2021; Willen et al., 2017). Structural discrimination therefore further compounds stress and limits coping resources for immigrants, making it challenging for them to manage everyday stressors effectively (Meyer et al., 2008; Quesada et al., 2011; Samari et al., 2021). Despite these structural inequities, immigrant communities are often cited as being incredibly resilient (Garcini et al., 2022). Central to contextualizing this resilience is understanding how immigrant communities resist systematic oppression, while simultaneously drawing attention to the structural harms and inequities that necessitate resilient traits and coping strategies.

Resilience emerged in the social science literature as a counter-narrative to discourses of risk, vulnerability, and social suffering that some scholars argued situated entire communities within a discourse of victimization (Panter-Brick, 2014). Rather than focusing on vulnerabilities and deficits, resilience-based approaches emphasize strengths, capabilities, resources, and transformative processes. Resilience is defined as a process of successful adaptation and development in the presence of adversity (Ungar, 2008). Such adaptation includes internal factors (psychological and physical health and well-being) as well as external factors (environmental adjustment). Resilience can therefore be understood through community and context-specific factors that incorporate multi-level, person-context interactions employed as responses to traumatic stimuli (Panter-Brick, 2014; Suslovic and Lett, 2023). Factors that promote resilience processes encompass individual, familial, community, and cultural attributes that reduce the risk of negative outcomes (Masten, 2004; Ungar, 2008). This includes both reactive capacity, where individuals cope and adapt to adverse conditions, and proactive capacity, in which individuals seek and create options (Ciaramella et al., 2022). As such, coping is a continuous and dynamic process that changes according to an individual’s capacity; external environmental factors related to scarcity, risk, oppression, and violence; sociodemographic characteristics; and the relationships and interactions between the individual and their social environments (Donnelly, 2002; Willen et al., 2022). However, much of the existing scholarship on resilience fails to acknowledge that it often arises as a response and adaptation to systematic and structural discrimination faced by oppressed populations (Cerdeña, 2023; Suslovic and Lett, 2023). This highlights a gap between research on resilience and evidence on the intersectional impacts of structural oppression on physical and mental health (Willen et al., 2022).

During the COVID-19 pandemic, the intersecting impacts of immigration and gender-related stressors presented unique challenges for racialized immigrant communities, particularly for immigrant women (Damle et al., 2022; Desai and Samari, 2020). Intersectionality theory highlights the concurrent and compounding impacts of multiple structures of inequality and systems of oppression faced by these communities (Crenshaw, 1991; Varcoe et al., 2019). The COVID-19 pandemic interacted and overlapped with context-specific drivers of vulnerability and marginalization, placing those with multiple or compounding vulnerabilities at greater risk of long-term consequences (Ryan and El Ayadi, 2020). The systemic oppression immigrant women faced before the pandemic contributed to gender differences within family systems during COVID-19, with immigrant women carrying a disproportionate burden of household responsibilities, including increased unpaid labor demands related to caregiving and family-focused household work (Power, 2020). Mothers, in particular, experienced heightened stress around parenting during the pandemic due to reduced productivity when working from home, increased domestic responsibilities, and increased caregiving responsibilities for children as a result of the closure of childcare institutions and the transition to online learning in school (Calarco et al., 2020; Hamel, 2020; Power, 2020). Throughout the pandemic, immigrant populations faced additional stressors, including fear of deportation, limited access to crucial social services, such as healthcare, ineligibility for national unemployment benefits, and exclusion from government relief packages (Wilson and Stimpson, 2020). The closure of schools and daycares further exacerbated gender inequalities in both paid and unpaid labor, as immigrant women took on more housework and childcare responsibilities at home (Haney and Barber, 2022). Balancing family and work became increasingly challenging, given the limited employment opportunities, financial strain, growing family responsibilities, and uncertainty arising from the suspension of services related to legal status (Trentin et al., 2023). The pandemic, therefore, magnified the pre-existing stressors and disparities faced by immigrant women, underscoring the need for an intersectional analysis of how structural racism and xenophobia, immigration, and gender-related stressors impact immigrant women’s health and well-being.

A syndemics framework helps explain how adverse social conditions stress a population, weaken its natural defenses, and expose it to a cluster of interacting diseases (Mendenhall, 2016). The COVID-19 pandemic exacerbated existing structural inequities, intensifying the challenges faced by immigrant women. These challenges encompassed severe economic and public health crises, limited access to healthcare and resources, and heightened psychological burdens (Garcini et al., 2022; Stolte et al., 2022). Before the pandemic, immigrant mothers were often responsible for caregiving, managing jobs, and fulfilling transnational care obligations. However, the combination of gendered roles and a collapsing economy disrupted the delicate balance between childcare and paid labor for immigrant mothers during the COVID-19 pandemic (Bruhn, 2022). This disruption further underscored the structural oppression experienced by immigrant communities, including discriminatory immigration policy, structural racism, and racialized violence, magnifying the overall impact of the pandemic on immigrant women and families. A syndemic perspective emphasizes the impact of historical factors, social determinants of health, and prevailing social justice issues as opposed to examining the COVID-19 pandemic as an isolated event (Grills et al., 2023). This approach offers a more comprehensive understanding of the complex interactions between the pandemic and pre-existing vulnerabilities, providing a critical lens for understanding the challenges faced by immigrant women during and beyond the COVID-19 pandemic.

The existing public health literature on immigrant populations in the U.S. focuses primarily on structural risk factors, like immigration policy and enforcement, and traumatic experiences, with significantly less research attention directed toward coping strategies and potential protective factors mitigating adversities and stressors (Cole et al., 2022; Novak et al., 2017; Panter-Brick, 2014; Samari et al., 2020). Additionally, while some research has explored coping and resilience among immigrant communities, it predominantly centers on Latinx populations and often fails to consider the intersectional identities and experiences of immigrant women from different countries of origin (Castañeda et al., 2015). This body of evidence explores mental health stressors and adaptations in response to discrimination, acculturative stress, and immigration-related stressors, with studies identifying cognitive and behavioral strategies, social support, faith, cultural pride, and transnational social networks as coping strategies used by immigrant populations and communities (Gonzalez et al., 2022; Lusk et al., 2021; Martin Romero et al., 2022; Rios Casas et al., 2020).

A limited number of studies have documented coping strategies among immigrant communities in the context of the COVID-19 pandemic. One qualitative study examined how Latina immigrant women navigated caregiving in the context of COVID-19-related school closures, highlighting how women continued to support their children’s education and socio-emotional well-being while confronting multiple levels of gendered racialized inequalities (Bruhn, 2022). Another mixed methods study, centered on Latinx communities in South Texas, identified behavioral and cognitive strategies, social support, and spirituality as primary coping mechanisms used by study participants to navigate stressors during COVID-19 (Garcini et al., 2022). While these studies provide valuable insights into Latinx immigrant experiences and coping strategies during the COVID-19 pandemic, they do not fully capture the intersectional and multidimensional coping experiences of immigrant women within the syndemic context of structural oppression and the COVID-19 pandemic. Notably, these studies lack the depth provided by perspectives from direct service providers working at community-based organizations (CBOs), who, due to their established relationships and trust within immigrant communities, offer additional insights and means of understanding how individual coping and resilience interplays with available resources, structures, and systems (Suva et al., 2022). This study aims to address these gaps by using an intersectional lens and syndemics framework to explore the individual, interpersonal, and community-level coping and adaptation strategies employed by immigrant women throughout the COVID-19 pandemic in response to stressors arising from the pandemic, immigration, gender expectations, and structural racism and xenophobia.

## 2. Methodology

### 2.1. Sources of data and eligibility criteria

This study consists of two samples: a) first- and second-generation cis-gender immigrant women of reproductive age living in New York City (NYC) (N = 44) and b) direct service providers providing healthcare or social services to immigrant women (N = 19). To be included in the immigrant women sample, individuals had to be 18–49 years old, reside in an NYC borough, identify as a cisgender woman, and be a first-or second-generation immigrant from Latin America, South or Southeast Asia, or the Middle East and North Africa, and be able to complete an interview in either English or Spanish. Women were recruited from these specific regions because these were the communities the study team had access to via partnerships with community-based organizations (CBOs).

To be eligible for the direct service provider sample, individuals had to work for an immigrant-serving organization providing family planning, social services, or health services; serve in a leadership role; provide services in an NYC borough; and be able to complete an interview in either English or Spanish.

### 2.2. Recruitment

Recruitment was facilitated through collaboration with immigrant-serving CBOs in the NYC metropolitan area that work with and provide services for immigrant communities, including health, education, social work, gender-based violence, legal, and financial services. Immigrant women meeting the eligibility criteria were invited to participate and additional participants were recruited via snowball sampling. The CBOs that facilitated recruitment for the immigrant women sample also facilitated initial outreach for service provider interviews, with additional service provider participants recruited via snowball sampling.

### 2.3. Data collection

Semi-structured in-depth interviews were conducted between September 2020–March 2021 by members of the research team who had received training in ethics and qualitative approaches to research among marginalized communities. A bilingual member of the research team conducted the interviews in English and Spanish. Interview guides were developed by the research team based on the literature on structural racism and migration and focused on immigrant women and direct service providers’ experiences, perceptions, and personal insights related to structural racism, xenophobia, and ways of coping with perceived challenges throughout the pandemic. The interview guides included questions about the specific personal beliefs, practices, relationships, resources, and services immigrant women drew from to cope with increased stress, anxiety, and fear and manage COVID-19-related challenges. Interviews were conducted in either English or Spanish, based on participant preference, and lasted approximately 45–60 min. Interviews were only conducted in English or Spanish due to language limitations among the study team. Due to restrictions on in-person activities during the time of data collection, all interviews took place virtually, either over the phone or through *Zoom*, based on participants’ preferences (Zoom, 2020).

The Columbia University Irving Medical Center’s Institutional Review approved the IRB application for this study in August 2020 (IRB-AAAT2404). Each participant received an information sheet about the study and provided verbal consent for participation and audio recording before the interview. All participants were given a $50 Amazon gift card for their participation in the study.

The study sample included 44 immigrant women and 19 direct service providers. The 44 immigrant women participants had a mean age of 33 years, with 50% from Latin America and about 52% with children (Table 1). The 19 direct service providers worked for CBOs (63%), healthcare clinics (21%), and family planning clinics (16%) providing gender-based violence (GBV) services (26%), immigration/social work services (37%), and sexual and reproductive health care (37%) (Table 2).

### 2.4. Data analysis

Interviews were audio recorded and recordings in English were transcribed using transcription software. Transcripts were manually compared to the audio recording for accuracy. A transcriber and a translator were hired to transcribe and translate interviews that were conducted in Spanish to English, and the materials were shared via a secure server to protect participant confidentiality. Transcripts were then deidentified and uploaded to *Dedoose* (Version 9.017), a qualitative data analysis platform, that was used to code and analyze the data (Dedoose, 2020). Separate coding and analytic processes were used for each sample. Codebooks were developed for both samples using a subset of transcripts and a line-by-line approach to identify themes and patterns. Additional thematic codes based on relevant literature were also integrated into the two codebooks. Several rounds of codebook revisions were conducted until there was a high level of agreement around the meaning and application of the codes. Data for both samples were analyzed using a constant comparative method to create codes, categories, and themes (Glaser and Strauss, 1967). Themes were developed through the coding process by identifying and interpreting patterns that ran within and across key categories related to participants’ perceptions of stressors and experiences of coping (Braun and Clarke, 2019).

### 2.5. Authors’ positionality

The social constructivist paradigm assumes multiple realities shaped by the position, standpoint, and values of the authors. Thus, we offer these findings as only one possible interpretation of these individuals’ experiences based on the authors’ experiences as educated, immigrant cisgender women. The first author is a U.S.-born, third generation immigrant, new to the study of racialization and migration, and reviewed and analyzed study materials after data collection, codebook development, and coding. The second author is a U.S.-born scholar who lives in Mexico City, is a mother, and has expertise in migration and gender-based research in Latin America and the United States; she conducted the Spanish and English language interviews. The senior author who led the study, is a U.S.-born ethnoracial minority scholar of immigrant parents, is a mother, and has been studying migration, racialization, and health for over a decade. Our racialized, nativity status and language skills put us in proximity to immigrant communities while our employment in an academic institution certainly creates distance. All authors worked as a team, having regular discussions to ensure the analysis was guided by their collective experience, knowledge, and expertise. It is likely, however, that our ethnoracial backgrounds, positions of privilege in an academic institution, nativity status, experiences as mothers, and gender influence our interpretations of the data, and we offer these findings as one possible perspective on coping and adaptation strategies employed by immigrant cisgender women in response to the syndemic of structural barriers.

## 3. Results

Based on the analysis of interviews with study participants, four distinct themes related to coping strategies and responses for immigrant women were identified: caregiving as a source of strength, leveraging resources, social connections, and community support.

### 3.1. Caregiving as a source of strength

Throughout the COVID-19 pandemic, participants’ coping strategies were largely shaped by their identities as women, mothers, caregivers, and immigrants and the resulting experiences, roles, and expectations related to those intersecting identities. Women described how they coped with disruptions to routines and social support brought on by the COVID-19 pandemic to meet the emerging needs of their families through adapting household dynamics, often taking on increased roles, responsibilities, and emotional labor around caregiving. Many participants described how difficult it was navigating isolation during COVID-19 as a mother, juggling their own health, well-being, work, and household responsibilities in addition to their children’s mental and emotional well-being, learning needs, and physical safety. As one mother who described having to quit her job caring for young children to protect her and her son’s health articulated, “It’s a lot, it’s hard work … it’s stressful to spend so much time confined, it’s made me cry, laugh, sad, a lot of things” (Dominican Republic, 43 years old). A service provider working at a community-based organization in Queens further emphasized their observation that, for many women, the increased stress and responsibilities around caregiving during the pandemic extended beyond children to other family members and in-laws:

now [women are] stuck at home taking care of the family and living with their in-laws … they all live in the same house. And that means a constant pressure … [feeling] like the in-laws’ needs must be satisfied. The kids’ needs must be satisfied. The husband’s needs must be satisfied. So women feel overwhelmed and they don’t have anywhere to go ….”

(General Services/Social Work Organization, Queens)

Although this increased care burden presented many challenges, women often cited their children and their responsibilities and obligations as mothers as a source of strength and motivation to persist. For example, one woman who has been living in the U.S. for 20 years but does not have citizenship status and was therefore ineligible for public benefits, when asked what gives her the strength to keep going and how she finds that kind of determination, responded: “My kids. I love my kids. And that makes me go” (India, 43 years old). Similarly, another participant described how, when she was feeling stressed and struggling to find sources of hope during the pandemic, she thought of her two children, “we have to continue fighting for our children” (Mexico, 45 years old). Service providers made similar observations about the resilience and perseverance of the mothers they worked with. As one provider articulated, “immigrant women who are parents … they fiercely do whatever they can for their children” (General Health/SRH Organization, Manhattan).

In addition to children providing sources of strength and motivation, several women also described appreciating how increased time at home during isolation allowed them to spend more time with their children and articulated how this helped to alleviate their own stress throughout the pandemic. When asked how she managed her stress, a young mother from Yemen with a new baby described:

I keep myself busy with my son … I like to see how he’s thinking, how he does stuff. I spend a lot of time with him and I’m just watching the stages of how a person grows and how they change and how they start talking, crawling … This is [how] I deal with my stress.

(Yemen, 26 years old)

Although many women framed children and caregiving responsibilities as sources of strength and motivation, participants also emphasized the enormous pressure and responsibility of being the primary caregiver and source of support for their family, often feeling alone and like a “one [wo]man army” (Korea, 46 years old). Several participants described feeling compelled to portray resilience and maintain an image of strength for the sake of their children, parents, and other family members. One single mother from Mexico with two children described her experience of finding strength out of necessity:

We want to throw in the towel, we want to give up, but I always think that we look at our children or our relatives who need us in Mexico and we get strength from I don’t know where and we keep on going forward.

(Mexico, 39 years old)

Similarly, another mother of two, also from Mexico, articulated how she and her partner tried to maintain a calm and happy facade to protect their children:

We’ve always tried to be happy and not seem worried as parents so the children don’t feel bad … but it makes us sad as parents, it makes us feel hopeless that the children might notice. So we always try to be calm at home and distract ourselves with little things for the kids to play and laugh.

(Mexico, 34 years old)

This intricate balance between outward strength and inner vulnerability was also observed by service providers working with immigrant women. Reflecting on these dualities, a service provider from an organization providing gender-based violence services in Manhattan shared:

I think that there’s incredible fragility. But there’s also an incredible survival instinct that I think we all could learn from … there’s a lot of fragility and inequity, but there’s also an incredible will to survive.

(GBV Organization, Manhattan)

In addition to gendered obligations around exhibiting strength, some participants articulated how experiences of discrimination shaped specific approaches to coping. Women discussed strategies of independence and self-reliance, articulating how they learned to stand up for themselves because they could not rely on anyone else (particularly the state) to protect and advocate for them. Some women described standing up for themselves against discrimination as a learned behavior and survival instinct in response to experiences of mistreatment and abuse. One participant from Bangladesh recounted an experience of discrimination and verbal abuse escalating to a threat of physical attack that happened while she was working at a restaurant a few years back. She described the fear she felt at that moment and how there were people all around her in the restaurant witnessing the verbal attack, yet “nobody stood up for [her]” (Bangladesh, 30 years old). She articulated what she took away from this experience:

I learned one thing. If no one stands up for you, you have to. You have to stand up for yourself. You have to take those steps that save you.

(Bangladesh, 30 years old)

This personal account aligns with insights shared by service providers who articulated how women’s individual coping strategies are shaped by structures and systems of exclusion and oppression that impose constraints on their ability to fulfill basic needs. As one service provider shared, when confronted with neglect, unmet needs and systemic failures from government and other entities, women are forced to adapt in order to survive:

We’re forced into being this image of strong black immigrant women like, yes, we are resilient. But at the same time, I think most people want to be soft and vulnerable and cared for, because when you’re pushed and when your needs aren’t met and you’re failed by government and by other structures and groups, you will do what you need to do to survive.

(General Services Organization, Manhattan)

Similarly, another young woman described how, “you need to stand up, not for yourself, but also for your family,” further emphasizing how women’s responses to discrimination are shaped by their perceived roles as caregivers and responsibilities related to protecting and prioritizing their family (Bangladesh, 23 years old).

Along this vein, several women described the emotional labor and mental toll of taking on their children’s stress, and in some cases, making sacrifices and prioritizing their children’s and family’s needs and well-being over their own. For example, a mother of three, originally from the Dominican Republic described prioritizing her children over everything “I try to make their world beautiful, even if mine isn’t” (Dominican Republic, 35 years old). This quotation highlights the responsibilities and commitments of motherhood and caregiving that, in some cases, come at a sacrifice to women’s well-being. Although women described “taking care of [their] children and do[ing] everything that’s possible for [their] family to take care of them” (Bangladesh, 23 years old) before the pandemic, many emphasized a dramatic shift in time for themselves that occurred during the pandemic, particularly related to children being home from school. Another woman similarly relayed how, with increased burdens and responsibilities around caregiving during the pandemic, “[women] are suffering with the time to [practice] self-care” (Korea, 46 years old). Service providers further emphasized the negative physical and mental health impacts of coping with these compounding responsibilities and stressors:

Stressors get piled on stressors and those do take a toll. Whether it’s shown or not, it’s taking a toll both emotionally and physically on people. And what you might not see today, you might see tomorrow. We know now that the effects of chronic stress lead to serious chronic health conditions

(General Health/SRH Organization, Manhattan).

Maintaining a delicate balance between recognizing immigrants’ resilience and acknowledging the substantial stress they endure, this service provider emphasized theneed for policies and services that support immigrants:

It’s really easy to say immigrants are just incredibly resilient … and they are. But I also don’t want to whitewash the sort of enormous stress that this poses. I don’t think we know completely what the damage from that stress is going to lead to. You have to remember a lot of undocumented families, also all the undocumented families didn’t get the relief packages that Americans got. So, they were also more disadvantaged from that standpoint as well, that financial standpoint. And that leads just enormously stressful, you know, whether you’re homeless or not. I mean, it’s just enormously stressful.

(General Health/SRH Organization, Manhattan).

### 3.2. Leveraging resources

Women described various activities that they engaged in to maintain a sense of purpose and control over their lives and their imagined futures, despite the disruptive impact of the pandemic and increased hostility and discrimination against immigrants. Recognizing the many ways in which the pandemic impaired women’s capacity for self-care, service providers described developed new programming:

We started doing virtual mental health sessions [to] guide people through breath work body exercises and centering themselves, because, again … we’re not able to able to really center ourselves as immigrant women because we [are] all consistently made to assume caregiving roles

(General Services Organization, Manhattan).

Many women recounted how, despite often limited resources, they prioritized self-care practices, including activities and hobbies such as meditation, exercising, reading, cooking, going for walks, writing, and spending time with pets. Participants emphasized how engaging in these activities eased anxiety and often served as a welcome distraction from stress related to the ongoing pandemic or the burdens of everyday discrimination. For example, one woman, when asked how she copes with stress and uncertainty, described: “I listen to music a lot. It calms me down and I try to watch movies … and exercise. Then probably cook or read I try everything to distract myself” (Bangladesh, 23 years old).

Additionally, although shifts to online activities were frequently cited as a barrier to accessing essential resources and care, women also highlighted how they navigated the online space, in some cases, identifying new opportunities that emerged. One woman described the utility of the Internet and Google as tools for increasing her ability to access health information and resources from home:

I love that I can get useful information on the Internet … I [am] always, researching and I started reading that book that it’s called My Insight and whatever I can get information from to help with my health care, with my mental health.

(Mexico, 43 years old)

Other women found that the shift to online courses facilitated by CBOs and other organizations during the pandemic provided them with new opportunities to advance their education and careers. One participant, for example, a 39-year-old from the Dominican Republic, discussed how before the pandemic, as a single mother of a child with special needs, she had limited options for attending night classes. However, during COVID-19, the newfound flexibility of virtual courses, paired with more time at home, opened new prospects.

I found someone who is teaching classes on how to promote your business on social media. I also found the English classes virtually; there are also organizations that have workshops for parents of children with special needs, so I’m attending workshops, support groups, and a little bit of everything.

(Dominican Republic, 39 years old)

Other women described new entrepreneurial activities they became involved in during the pandemic, such as building networks through online communities, providing online services (e.g., counseling), and other approaches to generating alternative sources of income (e.g., selling hand-crafted goods). For example, one young woman shared her experience of starting a small business making and selling paintings: “I started my own Etsy business and I started painting and a bunch of people wanted personal paintings and commissioned stuff. So that was fun. It was it was a great outlet for me” (Bangladesh (2nd gen), 24 years old).

### 3.3. Social connections

Moving beyond changes in behaviors and outlooks, many women described depending on their connections with family and friends as sources of comfort and emotional support mitigating the distress of loneliness and isolation. Service providers described how women often turned to their families for support, finding new ways to stay connected throughout the pandemic. As one provider observed:

I’ve seen a lot of people turning to family … they have this really strong support, even if they’re far away … they are trying to figure out ways to stay connected to those they feel separate from

(General Health/SRH Organization, Manhattan)

Women also described leveraging technology as a way of staying connected to family while physically distant. Staying in touch with family through WhatsApp group chats, texting, phone, and video calls helped women combat feelings of isolation related to both immigration and the pandemic. As one woman shared:

I’m Colombian and the family for us is really important. Even now with technology, it’s very easy to be connected with them … they were here to help me.

(Colombia, 30 years old)

Another participant described how communicating daily with her family back in the Dominican Republic through a WhatsApp group chat provided her with a sense of joy and support:

We have a WhatsApp group so I have my mother, my siblings, and my father and we spend the day there, sending jokes, talking, everything.

(Dominican Republic, 39 years old)

In alignment with accounts from immigrant women, service providers reiterated the importance of connections with family as a source of support, strength, and resilience for women:

They all seemed obviously stressed with the whole pandemic. However, they would talk about just being together as a family. And how it’s a source of strength to rely on family

(SRH Organization, Bronx)

While many women described seeking support from family, some women also expressed not wanting to reach out to their family for help so as not to cause stress or be perceived as a “burden.” In such cases, women often emphasized the importance of supportive social relationships and networks outside of the family. Participants described these relationships, particularly with other women, as critical in providing them with the opportunity to connect through shared experiences, talk freely, and express their emotions in ways they might not feel they were able to with their families. For example, when describing her experience participating in group video calls with other women, one participant recounted how these conversations got her through the pandemic:

If it hadn’t been for [these group video calls], I don’t know what would have become of me, because sometimes you can’t tell your family who is far away because you don’t want to worry them, “Oh I feel this way, I feel that way,” because they will worry since they’re far away.

(Dominican Republic, 43 years old)

This woman goes on to describe how beneficial conversations with other women were to her in easing her stress and anxiety throughout the pandemic: “It’s like a release … to be able to let it go … because when you let it go, it’s freeing … and it makes it easier” (Dominican Republic, 43 years old). Service providers similarly emphasized the value of social connections outside of the family for the immigrant women they worked with, particularly among women who were socially isolated and put their families’ needs above their own:

You have to have space … You have to have friends. You have to create boundaries. Space for yourself.

(General Services/Social Work Organization, Queens)

Another young woman from Bangladesh who had recently graduated from college and had no family in the U.S. described having regular check-ins with her friends throughout the pandemic:

I don’t know a single person who is not going through depression right now … I have friends all over and we talk, I talk to each and every one of them, we have weekly meetings, just to keep their mental health in check because everyone’s losing their sanity due to their personal relationships at home and domestic abuse or domestic violence and simply because of COVID and being woman, it’s been so difficult for all of these girls.

(Bangladesh, 25 years old)

As this participant explained, strong social networks and maintaining regular communications with friends were particularly critical for the women in her life throughout the pandemic as they were experiencing amplified mental health and gender-specific stressors.

Many CBOs recognized the importance of social connections for women beyond their immediate families. Service providers described their organizations’ gender- and culture-specific approaches to fostering connections and creating physical and virtual spaces for informal dialogue. For example, one service provider, whose organization primarily serves South Asian communities, described a weekly “Chai on Zoom” event their organization facilitated during the pandemic for women in the community to chat over biscuits and chai. This provider articulated how the “Chai on Zoom” events provide women with a valuable space to de-stress and connect with other women:

We talk about what we did, we talk about gossips happening in the community. Any recent news if someone is having a baby … or we talk about some kind of covid resources, so we make it fun. Fun in an educational way.

(General Services/Social Work Organization, Queens)

When speaking about the importance of social connections for immigrant women, another service provider emphasized how “those are what sustained [them]” throughout the pandemic, “socializing together, trying to use whatever they have to build community” (GBV Organization, Manhattan).

### 3.4. Community support

Beyond the support of friends and family networks and in the absence of government assistance and ineligibility for public benefits throughout the pandemic, immigrant women supported and were supported by their local communities and community-based organizations. Several women discussed their involvement in supporting and engaging their communities, emphasizing the importance of collective resilience in times of widespread material and social depravity. One service provider, who works at an organization providing support for people experiencing gender-based violence, articulated their observation of this sentiment among their immigrant clients: “there is a sense of community connectedness, of giving back” (GBV Organization, Manhattan). Although women’s activities and aspirations for supporting their local communities likely predated the pandemic, the role of community solidarity may have taken on a new or stronger meaning for women considering the extreme circumstances during COVID-19. One woman, who was born in the U.S. but whose parents are from Taiwan and Vietnam, illustrated this point, highlighting an example of how she copes with experiences of discrimination and hostility by finding sources of commonality across communities and ways of organizing and working together to ensure community needs are met:

I think seeking community with other people or trying to seek solidarity across different cultures where I’m like, oh OK, you experience your version of, you know, whatever. Me too, maybe together we can work together to figure it out.

(South Asia (2^nd^ gen), 33 years old)

For many women, CBOs played an integral role in fostering community to promote well-being and generate peer support among immigrant women beyond their existing family and social networks. One service provider highlighted their organization’s attention to building community among women, other immigrants, and the broader community:

We try to build community in that way … It’s always about also trying to build bridges and create a space for discussion with each other … with a lot of other women and other immigrants, but also not immigrants and men who are allies in their own ways and to also engage that discussion with them

(General Services Organization, Manhattan).

For some women, participating in community outreach efforts became an important source of coping with pandemic-generated isolation and other forms of mental health distress. One participant, for example, a 39-year-old from Mexico living in Queens with her four children, described how turning her gaze outward toward supporting her surrounding community helped her find an outlet from her internal struggles with fear, helplessness, and uncertainty of the future.

When I went out, I saw all those people who had no work and were sleeping in the street, long lines for food. So, thank God, my boss called me to work, and from what he gave me, there was hot food, and I gave it away to the people who live around here. I went out to the street to do that, it made me feel better, I forgot about my depression, I forgot about my fear, it became like – they are in need and I have to help them. I changed, like during covid I experienced what I had never experienced in my life, depression, sadness, fear, and feeling the need to help others …. I think it was a very, very difficult experience for many people, but at the same time, I think it brings out the best in you in that situation.

(Mexico, 39 years old).

As this participant discussed, despite facing constraining circumstances, helping others in her community became an opportunity for her to exercise agency and strive toward a better version of herself – leveraging her current situation to bring out the best in herself rather than acquiesce to the moral dread of physical and social stasis.

Service providers emphasized that support for one’s community in times of need can be a powerful source of agency and coping for immigrant women who have experienced or continue to contend with deep-seated structural inequities. One service provider, for example, commented that, although many migrants they work with experience significant inequity in their daily lives, “there’s also an incredible will to survive and find resources and share resources within the community” (GBV Organization, Manhattan).

Women also shared aspirations to support other migrant women and families who are experiencing challenges they once faced. As one woman who had experienced domestic abuse and struggled to pay rent and maintain secure housing for her daughters during the pandemic articulated: “In the future, I want to help women who have experienced what I’ve lived, from the heart, I want God to help me, to give me the opportunity, that’s one of my dreams” (42-year-old, Dominican Republic). Similarly, another woman, originally from Bangladesh, articulated her conviction in the importance of standing up for others experiencing discrimination, even if they did not stand up for her:

They couldn’t stand up for me. But, tomorrow if I was a customer and if this happened to somebody and if she didn’t have anyone to back her up. And if somebody is getting abusive like that, obviously I would stand up for her … How can we be selfish and just sit and watch?

(Bangladesh, 30 years old)

In addition to their contributions to their communities throughout the pandemic, many participants described benefitting from community-based social, material, and structural support and solidarity, facilitated through individuals and CBOs. With the shared understanding that many people’s basic needs were not being met throughout the pandemic, participants described different communities coming together to provide resources, food, and monetary assistance to those who needed it. Several participants described mutual aid programs where people who were in positions to do so agreed to split their stimulus checks and provide monetary support to immigrant families who lacked documentation and were excluded from public benefits. One woman from Bangladesh described how inspired she was by the inter-community support and solidarity she observed and experienced throughout the pandemic:

One of the best things that happened during COVID is everybody stood up for everyone. This was a test on humanity. You could tell who are those people who are there for you. And they were all strangers and they’re not related to me. They were all strangers. But the one thing that united us was humanity.

(Bangladesh, 30 years old)

While many women remarked on the increase of community support and solidarity as a positive outcome of the pandemic, some also raised concerns about the long-term sustainability of community support and reliance on CBOs in a post-pandemic world. One woman, a 32-year-old from the Dominican Republic, pointed to this complicated dynamic: “I have never seen so much support and so many things as in this year, I say wow, so much illness and so much death have to happen to have so much support, wow. How can it be, that you’re only moved when people have died, and people are sick?” (Dominican Republic, 32 years old).

## 4. Discussion

Findings from this study provide important insights into immigrant women’s coping strategies amid COVID-19 challenges and stressors within the context of structural inequities and oppression. The intersection of COVID-19 with existing structural inequities produced unique challenges and stressors for immigrant women related to the pandemic, caregiving responsibilities, immigration, and increased structural and social xenophobia (Damle et al., 2022; Fortuna et al., 2020). Despite facing overlapping stressors, many immigrants exhibited resilience coping mechanisms amid long-standing structural violence (J. P. Cerdeña, 2023; Garcini et al., 2022; Kalinowski et al., 2022). In this study, women described how their responses to challenges and stressors shaped their daily lives, attitudes, interactions, and communities. While coping strategies often fostered resilience, insights from service providers reveal how these strategies are shaped by structures, systems, and resource constraints. This emphasizes the deleterious effects of chronic exposure to stressors, highlighting a duality between strength and fragility.

Accounts from participants highlight how immigrant women navigated caregiving in the context of the health and economic crises of COVID-19, with limited access to social support. In line with evidence from past epidemics, during COVID-19, immigrant women disproportionately shouldered burdens related to social isolation, caregiving, and resource constraints, as they were increasingly called upon to care for and support their children, extended families in their countries of origin, and their communities (Bruhn, 2022; Connor et al., 2020; Laster Pirtle and Wright, 2021). While mothers in this study described joy related to their roles as mothers, they also articulated facing pressures around providing ideal health and learning environments for their children, sometimes resulting in feelings of stress, exhaustion, and anxiety (Rizzo et al., 2013).

In addition to bearing increased burdens, participants also developed creative responses to structural inequities that drew upon their intersectional identities as immigrant women and mothers. For example, several women described drawing strength and motivation from their caregiving responsibilities, particularly related to their children. In such cases, despite gendered expectations around caregiving and resource constraints during the pandemic, motherhood functioned as an important dimension of agency and motivation for women (Bruhn, 2022). Previous studies have identified immigrants’ resolve to create opportunities for their children and the next generation as a powerful motivator for enduring difficult circumstances and, in some cases, promoting resilience (Valdez et al., 2013). Among Latina migrant mothers in particular, recent scholarship has identified aspirations for children’s futures as enabling women to overcome trauma and ongoing adversity, and ultimately press onward, or “seguir adelante” (J. P. Cerdeña, 2023). Conceptualizing immigrant women’s pandemic care work with a deliberate emphasis on their power and agency as mothers underscores the significance of their intersectional identities as important socio-emotional resources, even during a time of crisis (Bruhn, 2022).

Along with these responsibilities, women described the emotional burden of feeling compelled to maintain an image of strength for their children and their families. Similar to the Superwoman Schema observed among Black women, this mode of coping reflects an internalized belief of needing to resist vulnerability while being obligated to help others, suppress emotions, and present an overall image of strength (Nelson et al., 2016). This mode of coping has been cited as having the potential to result in both adaptive and detrimental effects: contributing to the preservation of self, family, and community by instilling a sense of self-efficacy while simultaneously resulting in interpersonal relationship strain, negative physical health behaviors, and adverse psychological outcomes (Woods-Giscombe et al., 2019). Women alluded to these dual impacts as they described engaging in emotional labor, making sacrifices, and neglecting their own needs to ensure the health and well-being of their children and families.

In addition to negotiating gendered roles and responsibilities around caregiving, women also described centering their agency and maintaining aspirations for their futures. Many participants described leveraging resources and online platforms to cope with constrained circumstances and work towards their imagined futures. Such strategies align with prior research on building and preserving “moral agency,” characterized by intentions, aspirations, and access, in the context of crises where a sense of agency in everyday life may be eroded (Myers, 2016; Parson et al., 2022). Women identified new opportunities for growth and engagement that emerged throughout the pandemic, often facilitated through digital communities, resources, and information. Women discussed the role of community-based organizations as their main source of online courses, group forums, and other opportunities, emphasizing how these organizations helped them connect with other women and provided them with safe spaces, creative outlets, and new opportunities for personal and professional growth (Roels et al., 2022).

Beyond individual behaviors and outlooks, one of the most significant sources of support identified by participants was family. Although shifts to online activities and services have often been cited as a barrier for immigrant populations during the pandemic, immigrant women also leveraged technology in positive ways to stay connected to family while physically isolated during lockdown (Bastick and Mallet-Garcia, 2022). Women described how maintaining regular contact with family, both in the U.S. and in their country of origin, provided them with comfort and encouragement during difficult times. This aligns with prior research suggesting that cross-border ties and strong family relationships may serve as a buffer to stressors and discrimination experienced by immigrants (Bermudez and Mancini, 2013; Samari, 2016; Torres et al., 2016). Similarly, many women cited family and children as a source of strength and motivation to persist through challenging circumstances. Narratives from participants further highlight how family functioned as both a responsibility and system of support for immigrant women as they navigated challenges and stressors.

Results from this study suggest that some of the most impactful ways of coping with challenges and stressors throughout the COVID-19 pandemic were through harnessing community support, resources, and solidarity. During the pandemic, the U.S. government systematically excluded immigrant communities from essential resources and support, failing to meet their basic needs (Langellier, 2020; Page et al., 2020). As service providers articulated, this context of structural exclusion and resource scarcity shaped women’s coping strategies and experiences. In the absence of government support, many immigrant women described an inspiring surge in reciprocity and care both within and across communities. Women discussed how exerting agency through community-building and activism helped them cope with a diminished sense of agency in everyday life (Parson et al., 2022). Despite the surge in xenophobia and anti-immigrant attitudes since the start of the COVID-19 crisis, many women also expressed how different communities came together during the pandemic, sharing resources and providing essential support to those who were excluded and underserved by public programs (Esses and Hamilton, 2021).

Immigrant women and service providers described mutual aid networks that arose through the collaboration of individuals, communities, and organizations to provide support to those who needed it. This support helped to mitigate risk by providing resources that reduced exposure to hardships and allowed time, energy, and resources to be directed toward managing difficulties (Zilberstein, 2021). When describing support and solidarity across communities, participants emphasized the unprecedented care that emerged in parallel with the crisis of COVID-19 through the recognition of shared humanity (Littman et al., 2022). Results confirm how community support benefitted immigrant women in concrete ways, alleviating some of the material challenges they faced throughout the pandemic while also providing them with a valuable sense of hope, community connectedness, and faith in humanity amidst collectively experienced hardship.

While the findings from this study highlight promising coping strategies and support structures immigrant women used to manage stressors and challenges throughout the pandemic, it is critical to ensure that these stories of resilience and ability to persist through recurrent negative experiences do not obscure or normalize the structural inequities that produced them (Suslovic and Lett, 2023). As perspectives from direct service providers reveal, immigrant women’s coping experiences were a direct response to systematic and structural forms of discrimination and exclusion. In this study, participants’ social identities related to gender and immigration are inextricably linked to the stressors and inequities they experienced that necessitated coping in the first place. As direct service providers articulated, immigrant women’s coping strategies should draw attention to the harm-generating institutions, structures, and norms that produced them. Additionally, it is crucial to acknowledge the distress and lasting impact on mental health that the conditions of the COVID-19 pandemic, the sociopolitical climate around migration, related stressors, and coping have on immigrant communities. The detrimental physical and mental health effects arising from chronic and prolonged exposure to distressing and adverse experiences are well documented (Garcini et al., 2022). It has long been recognized that deterioration of health or the physiological burden imposed by stress is the product of weathering, or the cumulative health impact of repeated experiences with social or economic adversity and political marginalization (Geronimus et al., 2006).

While the coping strategies described in this study may have helped women navigate practical barriers, existing research underscores the adverse health effects of chronic exposure to stressors such as racism, financial instability, trauma, social inequalities, immigration-related stressors, and service barriers (Fortuna et al., 2020; Offidani-Bertrand, 2023). Service providers in this study highlighted how racial, social, and economic inequalities, intensified during the pandemic, contribute to toxic stress and cumulative disadvantage, impacting physical and mental health outcomes beyond the short-term efficacy of coping strategies. This perspective highlights the complexity of coping strategies as a mechanism of resilience while simultaneously being a potential source of dysfunction and a sign of harm. While some coping behaviors in response to harm may be linked to resilience in the short-term, they are often also associated with increased risk for stress-related illness with important consequences for migrants’ health and well-being (Garcini et al., 2022; Suslovic and Lett, 2023). Thus, as articulated by the immigrant women and service providers in this study, the conditions and stressors that demanded coping during COVID-19 highlight structural inequities and the duality of resilience and vulnerability among immigrant women.

### 4.1. Recommendations

Findings from this study highlight opportunities to promote the mental health and well-being of immigrant women and address the challenges they face due to structural oppression and heightened vulnerability during crises. As service providers working with immigrant communities are highly attuned to, structural barriers require structural solutions. As a first step, policymakers should pass inclusionary policy, especially in the face of crises, being mindful of the burden exclusion from policies like the 2020 CARES Act places on immigrant communities. As a second step, the service providers in this study recommend comprehensive, cross-sectoral support and service provision to alleviate burdens and enhance resilience. Strategies should adopt assets-based approaches that recognize community strengths and are responsive to the direct needs of immigrant communities. Additionally, strategies should build the capacity of immigrant-serving CBOs and incorporate insights from service providers working closely with and knowledgeable of these communities. Promising strategies that emerged from this study include improving access to affordable mental health services and other support services, developing communication strategies to better connect immigrants to existing community resources, and creating flexible, safe spaces for women to build social connections. It is critical to improve access to linguistically appropriate low- or no-cost mental health services for immigrant women, in a range of age-, and gender-specific modalities (e.g., one-on-one, child therapy, online support groups for women). Comprehensive, multifaceted support should be facilitated through community legal support, educational programs, financial support, and health services (Garcini et al., 2022). In addition, CBOs should be resourced to advertise existing programs and services to their constituents in a deeper and systematic way. This means forming partnerships between community-based organizations and other local health and social service providers and systems to streamline outreach and support. Finally, as emphasized by both women and direct service providers, flexible and safe community spaces to connect with other women are essential for building community and combatting social isolation. As we transition out of restrictions on in-person gatherings, CBOs can implement pre-pandemic strategies and continue to offer flexible options for virtual and online programming to facilitate participation and maximize engagement and accessibility. These approaches are essential to promoting the mental health and well-being of immigrant women and building trust with immigrant communities to address the impacts of both current and future crises.

### 4.2. Study limitations

This study is subject to certain limitations. Due to COVID-19 pandemic restrictions, in-person recruitment was limited and the virtual outreach approach that was employed may not have captured all available and willing participants. This could have affected the diversity of experiences and backgrounds of participants in the study. Additionally, participation in the study was limited to Spanish and English speakers. Importantly, the experiences of trans, nonbinary, and gender-expansive immigrants are missing. These populations likely experience coping and resilience in ways that are not captured in this study. Lastly, due to ongoing restrictions on in-person activities, all interviews had to be conducted virtually. This virtual format may have created digital barriers to participating and limited the accessibility of the study. However, this mode of data collection still generated rich qualitative data that provides valuable insights into the coping strategies immigrant women used in response to stressors and challenges throughout the pandemic.

## 5. Conclusion

Immigrant women were disproportionately impacted by the social, health, and economic burden of the COVID-19 pandemic in the United States (Garcini et al., 2022). This heightened vulnerability created a unique set of challenges and stressors related to the pandemic, immigration, and increased structural and social xenophobia. This study highlights multi-level coping strategies immigrant women used to respond to, alleviate, or reduce distress related to these compounding stressors. While coping strategies varied based on types of stressors as well as individual experiences and circumstances, women articulated how they responded to challenges they faced by drawing strength from understandings and expectations of motherhood and caregiving, leveraging resources, maintaining social connections, and seeking community support. Participants identified many approaches and strategies that promoted resilience, but the narratives of direct service providers also underscore the ways in which these coping strategies are connected to structures and systems of exclusion and oppression. Additionally, service providers highlighted the enduring mental and physical health consequences stemming from living in chronically stressful conditions and being forced to adapt to structural harm. A contextual understanding of both risk and protective factors among immigrant populations is critical to informing the allocation of resources and development of interventions to address the needs of these underserved communities while ultimately directing attention toward identifying and addressing the root causes of structural harm and vulnerability.

## Figures and Tables

**Table 1:** Descriptive characteristics (means or %) for immigrant women.

Key Variables	Immigrant Women (N = 44)	
	N	% or Mean (SD)
**Current Age (years)**	44	33.40 (7.26)
20–29	14	31.8%
30–39	21	47.7%
40–49	9	20.5%
**Region of Origin**		
Latin America	22	50.0%
South Asia	11	25.0%
East Asia	6	13.6%
Middle East & North Africa	5	11.4%
**Years in the US**	44	14.23 (9.66)
**Documented**	31	70.5%
**Employed**	27	61.4%
**Have any children**	23	52.3%
**# of Children**		
0	21	47.7%
1	6	13.6%
2+	17	38.6%

**Table 2:** Descriptive characteristics of direct service providers.

Key Variables	Direct Service Providers (N = 19)	
	N	%
**Type of Organization**		
Community-based	12	63%
Healthcare Clinic	4	21%
Family Planning Clinic	3	16%
**Service Type**		
Gender-based Violence	5	26%
Immigration/Social Work	7	37%
Sexual & Reproductive Health*	7	37%

Note:

* Three of the seven SRH service providers also provided primary care.

## References

[R1] Bastick Z, Mallet-Garcia M, 2022. Double lockdown: the effects of digital exclusion on undocumented immigrants during the COVID-19 pandemic. New Media Soc. 24 (2), 365–383. 10.1177/14614448211063185.

[R2] Bermudez JM, Mancini JA, 2013. Familias fuertes: family resilience among Latinos. In: Handbook of Family Resilience. Springer Science + Business Media, pp. 215–227. 10.1007/978-1-4614-3917-2_13.

[R3] Braun V, Clarke V, 2019. Reflecting on reflexive thematic analysis. Qualitative Research in Sport, Exercise and Health 11 (4), 589–597. 10.1080/2159676X.2019.1628806.

[R4] Bruhn S, 2022. “Me Cuesta Mucho”: Latina immigrant mothers navigating remote learning and caregiving during COVID-19. J. Soc. Issues 10.1111/josi.12546, 10.1111/josi.12546. PMC9538912 36249556

[R5] Calarco JM, Anderson E, Meanwell E, Knopf A, 2020. “Let’s not pretend it’s fun”: how COVID-19-related School and childcare Closures are damaging mothers’ well-being. SocArXiv. 10.31235/osf.io/jyvk4.

[R6] Castañeda H, Holmes SM, Madrigal DS, Young M-ED, Beyeler N, Quesada J, 2015. Immigration as a social determinant of health. Annu. Rev. Publ. Health 36 (1), 375–392. 10.1146/annurev-publhealth-032013-182419. 25494053

[R7] Cerdeña JP, 2023. Pressing Onward: the Imperative Resilience of Latina Migrant Mothers. Univ of California Press.

[R8] Ciaramella M, Monacelli N, Cocimano LCE, 2022. Promotion of resilience in migrants: a systematic review of study and psychosocial intervention. J. Immigr. Minority Health 24 (5), 1328–1344. 10.1007/s10903-021-01247-y. PMC9388436 34324124

[R9] Cole E, Su S, Diaz A, Zhang M, 2022. Social support and resilience among Burmese adolescent refugees: examining ethnic identity searching and belonging as moderators. Child. Youth Serv. Rev 142, 106647 10.1016/j.childyouth.2022.106647.

[R10] Connor J, Madhavan S, Mokashi M, Amanuel H, Johnson NR, Pace LE, Bartz D, 2020. Health risks and outcomes that disproportionately affect women during the Covid-19 pandemic: a review. Soc. Sci. Med 266, 113364 10.1016/j.socscimed.2020.113364. 32950924 PMC7487147

[R11] Crenshaw K, 1991. Demarginalizing the intersection of race and sex: a black feminist critique of antidiscrimination doctrine, feminist theory, and antiracist politics [1989]. In: Feminist Legal Theory, pp. 57–80. 10.4324/9780429500480-5. Routledge.

[R12] Damle M, Wurtz H, Samari G, 2022. Racism and health care: experiences of Latinx immigrant women in NYC during COVID-19. SSM - Qualitative Research in Health 2, 100094. 10.1016/j.ssmqr.2022.100094. 35578651 PMC9095080

[R13] Dedoose (9.017), 2020. In: [Computer Software]. SocioCultural Research Consultants (SCRC).

[R14] Desai S, Samari G, 2020. COVID-19 and immigrants’ access to sexual and reproductive health services in the United States. Perspect. Sex. Reprod. Health 52 (2), 69–73. 10.1363/psrh.12150. 32530526 PMC7307065

[R15] Donnelly TT, 2002. Contextual analysis of coping: implications for immigrants’ mental health care. Issues Ment. Health Nurs 23 (7), 715–732. 10.1080/01612840290052848. 12227837

[R16] Esses VM, Hamilton LK, 2021. Xenophobia and anti-immigrant attitudes in the time of COVID-19. Group Process. Intergr. Relat 24 (2), 253–259. 10.1177/1368430220983470.

[R17] Esterline C, Batalova J, 2022. Frequently Requested Statistics on Immigrants and Immigration in the United States. Migrationpolicy.Org. https://www.migrationpolicy.org/article/frequently-requested-statistics-immigrants-and-immigration-united-states.

[R18] Fortuna LR, Tolou-Shams M, Robles-Ramamurthy B, Porche MV, 2020. Inequity and the disproportionate impact of COVID-19 on communities of color in the United States: the need for a trauma-informed social justice response. Psychological Trauma: Theory, Research, Practice, and Policy 12 (5), 443. 10.1037/tra0000889. 32478545 PMC8243721

[R19] Garcini LM, Cadenas G, Domenech Rodríguez MM, Mercado A, Campos L, Abraham C, Silva M, Paris M, 2022. Lessons learned from undocumented Latinx immigrants: how to build resilience and overcome distress in the face of adversity. Psychol. Serv 19, 62–71. 10.1037/ser0000603. 34807667 PMC8989674

[R20] Geronimus AT, Hicken M, Keene D, Bound J, 2006. “Weathering” and age patterns of allostatic load scores among blacks and whites in the United States. Am. J. Publ. Health 96 (5), 826–833. 10.2105/AJPH.2004.060749. PMC1470581 16380565

[R21] Glaser BG, Strauss AL, 1967. The Discovery of Grounded Theory: Strategies for Qualitative Research. Aldine Publishing. http://lib.myilibrary.com/browse/open.asp?id=620109&entityid=https://idp.brunel.ac.uk/entity.

[R22] Gonzalez LM, Mejia Y, Kulish A, Stein GL, Kiang L, Fitzgerald D, Cavanaugh A, 2022. Alternate approaches to coping in latinx adolescents from immigrant families. J. Adolesc. Res 37 (3), 353–377. 10.1177/0743558420914083.

[R23] Grills C, Carlos Chavez FL, Saw A, Walters KL, Burlew K, Randolph Cunningham SM, Rosario CC, Samoa R, Jackson-Lowman H, 2023. Applying culturalist methodologies to discern COVID-19’s impact on communities of color. J. Community Psychol 51 (6), 2331–2354. 10.1002/jcop.22802. 35102549 PMC9015500

[R24] Hamel L, 2020. Is There a Widening Gender Gap in Coronavirus Stress? KFF. https://www.kff.org/policy-watch/is-there-widening-gender-gap-in-coronavirus-stress/.

[R25] Haney TJ, Barber K, 2022. The extreme gendering of COVID-19: household tasks and division of labour satisfaction during the pandemic. Canadian Review of Sociology/Revue Canadienne de Sociologie 59 (S1), 26–47. 10.1111/cars.12391. 35946961 PMC9537987

[R26] Kalinowski J, Wurtz H, Baird M, Willen SS, 2022. Shouldering the load yet again: black women’s experiences of stress during COVID-19. SSM - Mental Health 2, 100140. 10.1016/j.ssmmh.2022.100140. 35974954 PMC9371978

[R27] Langellier BA, 2020. Policy recommendations to address high risk of COVID-19 among immigrants. Am. J. Publ. Health 110 (8), 1137–1139. 10.2105/AJPH.2020.305792. PMC7349431 32584591

[R28] Laster Pirtle WN, Wright T, 2021. Structural gendered racism revealed in pandemic times: intersectional approaches to understanding race and gender health inequities in COVID-19. Gend. Soc 35 (2), 168–179. 10.1177/08912432211001302.

[R29] Littman DM, Boyett M, Bender K, Dunbar AZ, Santarella M, Becker-Hafnor T, Saavedra K, Milligan T, 2022. Values and beliefs underlying mutual aid: an exploration of collective care during the COVID-19 pandemic. J. Soc. Soc. Work. Res 13 (1), 89–115. 10.1086/716884.

[R30] Lusk M, Terrazas S, Caro J, Chaparro P, Puga Antúnez D, 2021. Resilience, faith, and social supports among migrants and refugees from Central America and Mexico. J. Spirituality Ment. Health 23 (1), 1–22. 10.1080/19349637.2019.1620668.

[R31] Martin Romero MY, Gonzalez LM, Stein GL, Alvarado S, Kiang L, Coard SI, 2022. Coping (together) with hate: strategies used by Mexican-origin families in response to racial–ethnic discrimination. J. Fam. Psychol 36, 3–12. 10.1037/fam0000760. 33661688 PMC8896309

[R32] Masten AS, 2004. Regulatory processes, risk, and resilience in adolescent development. Ann. N. Y. Acad. Sci 1021 (1), 310–319. 10.1196/annals.1308.036. 15251901

[R33] McKnight-Eily LR, 2021. Racial and Ethnic Disparities in the Prevalence of Stress and Worry, Mental Health Conditions, and Increased Substance Use Among Adults during the COVID-19 Pandemic—United States, April and May 2020. MMWR. Morbidity and Mortality Weekly Report, vol. 70. 10.15585/mmwr.mm7005a3. PMC7861483 33539336

[R34] Mendenhall E, 2016. Syndemic Suffering: Social Distress, Depression, and Diabetes Among Mexican Immigrant Women. Routledge.

[R35] Meyer IH, Schwartz S, Frost DM, 2008. Social patterning of stress and coping: does disadvantaged social statuses confer more stress and fewer coping resources? Soc. Sci. Med 67 (3), 368–379. 10.1016/j.socscimed.2008.03.012. 18433961 PMC2583128

[R36] Myers NAL, 2016. Recovery stories: an anthropological exploration of moral agency in stories of mental health recovery. Transcult. Psychiatr 53 (4), 427–444. 10.1177/1363461516663124. 27578861

[R37] Nelson T, Cardemil EV, Adeoye CT, 2016. Rethinking strength: black women’s perceptions of the “strong black woman” role. Psychol. Women Q 40 (4), 551–563. 10.1177/0361684316646716.

[R38] Novak NL, Geronimus AT, Martinez-Cardoso AM, 2017. Change in birth outcomes among infants born to Latina mothers after a major immigration raid. Int. J. Epidemiol 46 (3), 839–849. 10.1093/ije/dyw346. 28115577 PMC5837605

[R39] Offidani-Bertrand C, 2023. “It unleashed all the worries we tried to calm down”: the Trump administration’s impact on the mental health of immigrant communities. SSM - Mental Health 3, 100207. 10.1016/j.ssmmh.2023.100207.

[R40] Page KR, Venkataramani M, Beyrer C, Polk S, 2020. Undocumented U.S. Immigrants and covid-19. N. Engl. J. Med 382 (21), e62. 10.1056/NEJMp2005953. 32220207

[R41] Panter-Brick C, 2014. Health, risk, and resilience: interdisciplinary concepts and applications. Annu. Rev. Anthropol 43, 431–448. 10.1146/annurev-anthro-102313-025944.

[R42] Parson NC, Wurtz HM, Lowrey M, Santos C, 2022. “Life will go on with the beauty of the roses”: the moral dimensions of coping with distress through autobiographical writing during Covid-19. SSM - Mental Health 2, 100156. 10.1016/j.ssmmh.2022.100156.

[R43] Power K, 2020. The COVID-19 pandemic has increased the care burden of women and families. Sustain. Sci. Pract. Pol 16 (1), 67–73. 10.1080/15487733.2020.1776561.

[R44] Quesada J, Hart LK, Bourgois P, 2011. Structural vulnerability and health: latino migrant laborers in the United States. Med. Anthropol 30 (4), 339–362. 10.1080/01459740.2011.576725. 21777121 PMC3146033

[R45] Rios Casas F, Ryan D, Perez G, Maurer S, Tran AN, Rao D, Ornelas IJ, 2020. “Se vale llorar y se vale reír”: Latina immigrants’ coping strategies for maintaining mental health in the face of immigration-related stressors. Journal of Racial and Ethnic Health Disparities 7 (5), 937–948. 10.1007/s40615-020-00717-7. 32040841 PMC7416525

[R46] Rizzo KM, Schiffrin HH, Liss M, 2013. Insight into the parenthood paradox: mental health outcomes of intensive mothering. J. Child Fam. Stud 22 (5), 614–620. 10.1007/s10826-012-9615-z.

[R47] Roels NI, Estrella A, Maldonado-Salcedo M, Rapp R, Hansen H, Hardon A, 2022. Confident futures: community-based organizations as first responders and agents of change in the face of the Covid-19 pandemic. Soc. Sci. Med 294, 114639 10.1016/j.socscimed.2021.114639. 34998135 PMC8683095

[R48] Ryan NE, El Ayadi AM, 2020. A call for a gender-responsive, intersectional approach to address COVID-19. Global Publ. Health 15 (9), 1404–1412. 10.1080/17441692.2020.1791214. 32633628

[R49] Samari G, 2016. Cross-border ties and Arab American mental health. Soc. Sci. Med 155, 93–101. 10.1016/j.socscimed.2016.03.014. 26999416 PMC4819968

[R50] Samari G, Catalano R, Alcalá HE, Gemmill A, 2020. The Muslim Ban and preterm birth: analysis of U.S. vital statistics data from 2009 to 2018. Soc. Sci. Med 265, 113544 10.1016/j.socscimed.2020.113544. 33261902 PMC7738384

[R51] Samari G, Nagle A, Coleman-Minahan K, 2021. Measuring structural xenophobia: U. S. state immigration policy climates over ten years. SSM - Population Health 16, 100938. 10.1016/j.ssmph.2021.100938. 34660879 PMC8503659

[R52] Sangaramoorthy T, Carney MA, 2021. Immigration, mental health and psychosocial well-being. Med. Anthropol 40 (7), 591–597. 10.1080/01459740.2021.1931174. 34107226

[R53] Stolte A, Nagy GA, Zhan C, Mouw T, Merli MG, 2022. The impact of two types of COVID-19-related discrimination and contemporaneous stressors on Chinese immigrants in the US South. SSM - Mental Health 2, 100159. 10.1016/j.ssmmh.2022.100159. 36188193 PMC9509533

[R54] Suslovic B, Lett E, 2023. Resilience Is an Adverse Event: A Critical Discussion of Resilience Theory in Health Services Research and Public Health. Community Health Equity Research & Policy. 10.1177/2752535X231159721, 2752535X231159721. PMC10919062 36856261

[R55] Suva C, Liu J, Sigurdson E, Torio JE, Benson OG, 2022. A case study of community-based, cross-sectoral crisis response to the COVID-19 pandemic: serving racialized immigrant communities. Global Social Welfare 9 (3), 193–202. 10.1007/s40609-022-00223-0. 35313616 PMC8927743

[R56] Torres JM, Alcántara C, Rudolph KE, Viruell-Fuentes EA, 2016. Cross-border ties as a source of risk and resilience: do cross-border ties moderate the relationship between migration-related stress and psychological distress among Latino migrants in the United States? J. Health Soc. Behav 57 (4), 436–452. 10.1177/0022146516667534. 27803264 PMC5444403

[R57] Trentin M, Rubini E, Bahattab A, Loddo M, Della Corte F, Ragazzoni L, Valente M, 2023. Vulnerability of migrant women during disasters: a scoping review of the literature. Int. J. Equity Health 22 (1), 135. 10.1186/s12939-023-01951-1. 37481546 PMC10362632

[R58] Ungar M, 2008. Resilience across cultures. Br. J. Soc. Work 38 (2), 218–235. 10.1093/bjsw/bcl343.

[R59] Valdez C, Valentine J, Padilla B, 2013. “Why we stay”: immigrants’ motivations for remaining in communities impacted by anti-immigration policy. Cult. Divers Ethnic Minor. Psychol 19, 279–287. 10.1037/a0033176. PMC3721425 23875853

[R60] Varcoe C, Browne A, Blanchet Garneau A, 2019. Beyond stress and coping: the relevance of critical theoretical perspectives to conceptualising racial discrimination in health research. Health Sociol. Rev 28 (3), 245–260. 10.1080/14461242.2019.1642124.

[R61] Willen SS, Knipper M, Abadía-Barrero CE, Davidovitch N, 2017. Syndemic vulnerability and the right to health. Lancet 389 (10072), 964–977. 10.1016/S0140-6736(17)30261-1. 28271847

[R62] Willen SS, Williamson AF, Walsh CC, Hyman M, Tootle W, 2022. Rethinking flourishing: critical insights and qualitative perspectives from the U.S. Midwest. SSM - Mental Health 2, 100057. 10.1016/j.ssmmh.2021.100057. 34961852 PMC8694651

[R63] Wilson FA, Stimpson JP, 2020. US policies increase vulnerability of immigrant communities to the COVID-19 pandemic. Annals of Global Health 86 (1), 57. 10.5334/aogh.2897. PMC7292131 32566486

[R64] Woods-Giscombe CL, Allen AM, Black AR, Steed TC, Li Y, Lackey C, 2019. The giscombe superwoman Schema questionnaire: psychometric properties and associations with mental health and health behaviors in African American women. Issues Ment. Health Nurs 40 (8), 672–681. 10.1080/01612840.2019.1584654. 31081707 PMC7220093

[R65] Zilberstein K, 2021. Debate: coping and resilience in the time of COVID-19 and structural inequities. Child Adolesc. Ment. Health 26 (3), 279–280. 10.1111/camh.12484. 34196096 PMC8444833

[R66] Zoom (5.12.2 (11434)). (2020). [Computer Software]. Zoom Video Communications, Inc.

